# Potential Impact of Intermittent Preventive Treatment (IPT) on Spread of Drug-Resistant Malaria

**DOI:** 10.1371/journal.pmed.0030141

**Published:** 2006-04-04

**Authors:** Wendy Prudhomme O'Meara, David L Smith, F. Ellis McKenzie

**Affiliations:** **1**Fogarty International Center, National Institutes of Health, Bethesda, Maryland, United States of America; Mahidol UniversityThailand

## Abstract

**Background:**

Treatment of asymptomatic individuals, regardless of their malaria infection status, with regularly spaced therapeutic doses of antimalarial drugs has been proposed as a method for reducing malaria morbidity and mortality. This strategy, called intermittent preventive treatment (IPT), is currently employed for pregnant women and is being studied for infants (IPTi) as well. As with any drug-based intervention strategy, it is important to understand how implementation may affect the spread of drug-resistant parasites. This is a difficult issue to address experimentally because of the limited size and duration of IPTi trials as well as the intractability of distinguishing the spread of resistance due to conventional treatment of malaria episodes versus that due to IPTi when the same drug is used in both contexts.

**Methods and Findings:**

Using a mathematical model, we evaluated the possible impact of treating individuals with antimalarial drugs at regular intervals regardless of their infection status. We translated individual treatment strategies and drug pharmacokinetics into parasite population dynamic effects and show that immunity, treatment rate, drug decay kinetics, and presumptive treatment rate are important factors in the spread of drug-resistant parasites. Our model predicts that partially resistant parasites are more likely to spread in low-transmission areas, but fully resistant parasites are more likely to spread under conditions of high transmission, which is consistent with some epidemiological observations. We were also able to distinguish between spread of resistance due to treatment of symptomatic infections and that due to IPTi. We showed that IPTi could accelerate the spread of resistant parasites, but this effect was only likely to be significant in areas of low or unstable transmission.

**Conclusions:**

The results presented here demonstrate the importance of considering both the half-life of a drug and the existing level of resistance when choosing a drug for IPTi. Drugs to which little or no resistance exists are not advisable for IPT in high-transmission areas, but IPTi is not likely to significantly impact the spread of highly resistant parasites in areas where partial resistance is already established. IPTi is more likely to accelerate the spread of resistance in high-transmission areas than is IPT in adults (i.e., pregnant women).

## Abstract 2

A new malaria model predicts that deploying intermittent preventive treatment (IPT) in infancy could accelerate the spread of resistant parasites, but that this effect would be significant only in areas of low or unstable transmission.

## Introduction

Rapidly spreading resistance to antimalarial drugs is threatening the ability to treat malaria effectively in nearly every malaria-endemic region of the globe. The least expensive and safest treatment, chloroquine, has been replaced as the first-line drug in many countries due to increasing prevalence of resistant parasites. Resistance to the cheapest alternative, sulfadoxine-pyrimethamine (SP), is threatening the viability of this treatment as well. New and affordable pharmaceuticals are needed, but to safeguard their usefulness as well as to extend the life of existing drugs such as SP, the spread of drug resistance must be minimized. It is critical to understand how patterns of drug use and the epidemiological context of drug deployment contribute to the emergence and spread of drug resistance in order to protect the efficacy of antimalarial drugs.

Genetic mutations that confer some level of protection against a drug are thought to arise randomly during replication [ 1]. Selection occurs when the parasites in an infection are exposed to a drug: resistant mutants have a survival advantage over parasites without the mutation. The mutations can spread if gametocytes carrying the advantageous mutation are transmitted to a feeding mosquito.

Typically, high-level resistance to a particular drug requires multiple mutations that accumulate gradually over several parasite generations. For example, increasing resistance to SP has been correlated with the stepwise acquisition of specific point mutations [ 2, 3]. Parasites with different sensitivities are able to grow at different concentrations of the drug. Fully resistant parasites will survive a full therapeutic dose, whereas partially resistant parasites will be selected for at intermediate concentrations that are still inhibitory for fully sensitive parasites. The parasite-drug interaction manifests clinically as reappearance of a detectable density of parasites within days after treatment (partially resistant) or infections that do not respond to treatment at all (fully resistant).

**Figure 1:**
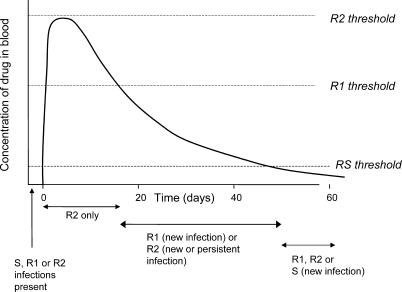
Pharmacokinetics of Drug Resistance Concentration of drug in the bloodstream declines over time and falls below threshold levels permissive for growth of parasites with varying degrees of resistance. Dashed lines represent permissive thresholds for RS, R1, and R2 parasites. Adapted from Hastings et al. [ 11]. RS, drug-sensitive parasites; R1, partially resistant parasites; R2, fully resistant parasites.

The pharmacokinetic properties of drug elimination from the bloodstream determine the window of selection for resistance mutations ( Figure 1). Drugs with long half-lives, such as SP, provide longer protection against infection, but they also increase parasite exposure to subtherapeutic drug concentrations, and thus selection for resistant mutants. Infections that appear during the gradual decay of SP in the bloodstream of a treated patient are more likely to be pyrimethamine-resistant than those that appear after the drug has cleared [ 4].

Drug treatment and immune mechanisms act synergistically to clear an infection. Even at permissive concentrations of drug, antiparasite immunity can prevent resistant parasites from proliferating [ 5, 6]. It has been proposed that the spread of drug resistance is driven by the greater survival and transmission of resistant versus sensitive phenotypes during the time between administration of the drug and immune clearance of the infection [ 7]. That interval is maximized when individuals with little or no parasite-specific immunity, such as infants, are given antimalarial drugs.

Drug use patterns, drug pharmacokinetics, and antimalarial immunity all impact the spread of drug-resistant parasites. These three factors converge in a unique synergy in infants undergoing intermittent preventive treatment (alternatively, intermittent presumptive therapy), or IPT, an intervention that involves administering antimalarial drugs to individuals on a fixed schedule, regardless of disease or infection status [ 8– 10]. During IPT, blood concentrations of the drug are not maintained continuously at levels high enough to clear and prevent infection; drug titers are expected to initially rise and then subside between treatments. When these regular fluctuations of drug titer occur in infants, whose immunity is not robust or specific enough to control infections, new infections that emerge from the liver between treatments are exposed to conditions that are ideal for selectively spreading resistant parasites [ 4, 11]. Moreover, because *Plasmodium* blood-stage densities in infants and children are typically orders of magnitude higher than in adolescents and adults, it is much more likely that resistant infections will reach high densities and increase their probability of being transmitted [ 12]. In at least two trials of IPT for infants (IPTi), there were significant increases in the frequency of drug-resistant infections in the treated group relative to the control group [ 13, 14]. The situation is exacerbated still further if infants and children represent disproportionate reservoirs of transmission in malaria-endemic settings, as some field studies suggest [ 15– 17]. Thus several lines of reasoning argue that infants and children have a particularly significant role in the community-wide spread of drug-resistant parasites.

In order to understand how drug use, pharmacokinetics, immune status, and transmission intensity may act synergistically on the spread of drug-resistant malaria, we developed a model that combines drug use patterns with parasite fitness to predict the spread of resistance. Resistance to antimalarial drugs is not a simple switch but requires an accumulation of genetic changes that confer increasing levels of resistance. Hastings et al. [ 11] developed one of the first models of antimalarial resistance that incorporated drug pharmacokinetics into an analysis of the spread of drug resistance; the authors did so by allowing parasites to be either drug sensitive, resistant to low levels of drug (partially resistant), or resistant to full therapeutic doses (fully resistant). Their model describes drug use as a single parameter applied uniformly to a homogenous population, leaving aside transmission intensity and immunity. Here we develop explicit relationships between transmission intensity, immunity, and drug policy in an epidemiological model that allows these complicated and interdependent factors to be included simultaneously, but evaluated independently.

## Methods

Our analysis of the spread of drug-resistant parasites uses a composite model of human disease and treatment and parasite fitness. The human population model tracks the total dosing with antimalarial drugs, considering infection, treatment, temporary immunity to reinfection, and the development of immunity after previous exposure to infection. The parasite population model determines which potential hosts are available for infection by parasites with different levels of drug sensitivity.

### Human Dosing Model

Immunity to malaria develops slowly, after repeated infection [ 18], and can affect the emergence and spread of drug resistance. To model immunity, we have adapted the Garki model [ 19], in which individuals who acquire some immunity graduate to a set of compartments for infection status and treatment that parallels those who lack it.

Individuals are born nonimmune and experience a cycle of infection, treatment or clearance of parasites, and short-lived immunity immediately following an episode, until graduating to a semi-immune state. Semi-immune individuals experience the same cycle, but with a higher probability of carrying an asymptomatic infection and a longer interval between each infection. The graduation from nonimmune to semi-immune depends, at least in part, on the number of attacks, and thus on the frequency of infectious bites. Intervention strategies, such as chemoprophylaxis and IPTi, may be applied differentially to different age groups (or, here, groups with different immune status).

Specifically, infants are born susceptible (compartment S) to malaria infection at a constant rate *(μ),* and there is a constant death rate from each compartment ( *μ*X, where X = S, A, P, and the other compartments [ Table 1]). The rate at which infectious mosquitoes bite humans is known as the entomological inoculation rate *(Λ)*. A fraction *(λ)* of susceptible nonimmunes who are bitten will become symptomatic and have a probability *(p)* of being treated. Symptomatic treated individuals are designated by D. The remaining infected individuals are untreated, designated by A, and include those who are asymptomatic as well as those with symptomatic infections that will go untreated. Some of the untreated individuals will later become symptomatic, either by receiving a new infectious bite *(dΛ)* or due to other precipitating factors *(ν),* and have a probability *(p)* of being treated. In the treated, symptomatic individuals (D), the waiting time between the onset of symptoms and the time when the drug titer becomes high enough to clear the infection is 1/ *a* d, at which point they enter a new class T _1_. Over a period of 1/ *r*_1_ d, which is determined by the pharmacokinetic properties of the drug, the drug concentration declines to a level permissive to partially resistant parasites (T _1_′). The drug concentration falls to zero over an additional period of 1/ *r*_2_ d. After a treated clinical attack, antimalarial immunity (P) develops and provides protection from a new infection for 1/ *w* d. Thus, the concentration of drug in the blood following treatment declines as a stepwise process until all of the drug is cleared (D → T _1_ → T _1_′ → P). Untreated asymptomatic and symptomatic infections (A) are either cleared quickly and spontaneously with no subsequent protection *(σ)* or they are cleared more slowly by immune mechanisms *(g)* resulting in protection from reinfection (P) for 1/ *w* d.

**Table 1:**
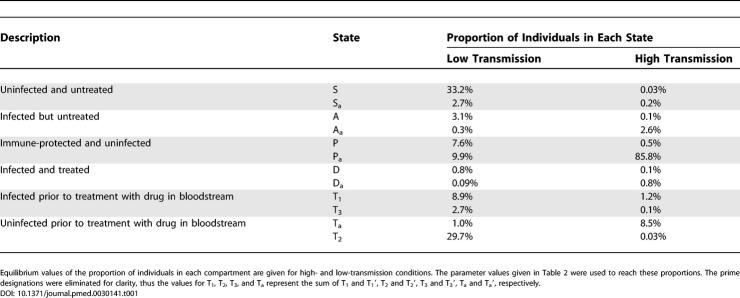
Compartments of Human Dosing Model Categorized by Treatment, Infection, and Immune Status

Susceptible, nonimmune individuals (S) *,* those with untreated infections (A), and those with immune protection (P) may undergo periodic drug dosing during IPTi at a rate, *c,* and they progress through a stepwise decay of blood drug concentration similar to those treated for symptoms. A dose of antimalarial drug during IPTi clears any existing, asymptomatic, drug-sensitive infection and prevents reinfection by partially or fully sensitive parasites for a period of 1/ *r*_1_ d. Between 1/ *r*_1_ and 1/ *r*_1_ + 1/ *r*_2_ d, the drug concentration drops to a level that is still active against fully sensitive parasites, but is permissive for infection by partially resistant parasites (i.e., T _x_ → T _x_′, where *x* = 1, 2, 3, *a*). Susceptible nonimmunes (S) become treated nonimmunes (T _2_) after a dose of drug during IPTi, then decay to a level of lower protection (T _2_′), and then return to the susceptible class (S → T _2_ → T _2_′ → S). Those with untreated infections *(A)* who are treated through IPTi (T _3_) become partially protected (T _3_′), and have a probability *(b)* of acquiring immune protection as a result of the infection (A → T _3_ → T _3_′ → S or A → T _3_ → T _3_′ → P). Immune-protected individuals (P) may also be treated through IPTi, but IPTi does not affect the development or loss of immunity. We assume that nonimmunes who arrive for IPT treatments with clinical symptoms of malaria are diagnosed and treated (D). Semi-immune status is acquired with some probability after clearing an infection, at a rate that is proportional to entomological inoculation rate (EIR) *(γΛ)*. Immunity to reinfection (P _a_) persists for a period of 1/ *w*′ d, after which susceptibility (S _a_) to infection returns. A greater proportion (1 − *λ*′) of those who are infected maintain an asymptomatic infection or develop symptoms but go untreated (A _a_), but a small fraction ( *λ*′) become ill and have a probability *(p)* of being treated (D _a_) with an antimalarial drug. Treatment leads to parasite clearance and a gradual decline of drug titers, as described above (T _a_ → T _a_′). Asymptomatic, infected individuals may become ill for various reasons ( *ν*′), but not due to superinfection, and have a probability *(p)* of being treated for malaria.

A summary of the compartments with respect to their disease and treatment status can be found in Table 1, along with the equilibrium values of the proportion of individuals in each state at any given time. The equations describing disease and drug dosing in the human host are in Text S1.

### Model of Parasite Fitness

The impact of infection and treatment described above on the spread of resistance in these models can be measured by determining how effectively parasites with different levels of resistance compete under different drug usage scenarios. To do this, we determined the relative fitness of different parasite populations, building on the concepts developed by Hastings et al. [ 11]. The fitness of one parasite population relative to another is directly proportional to the ratio of the duration of infection, number of human hosts available for infection, and transmission efficiency of each population. We assumed that there is no difference in transmission efficiency between the sensitive and resistant populations and that the mutations conferring resistance have negligible impact on parasite fitness in the absence of drug (i.e., there is no cost of resistance); therefore the fitness of a partially or fully resistant parasite relative to the sensitive parasite is the ratio of their average durations of infection multiplied by the ratio of the number of hosts available to each to infect. The assumption of equivalent transmission may not always hold true, especially where drug resistance appears to correlate with increased gametocyte production and, possibly, transmission [ 20, 21].

**Figure 2:**
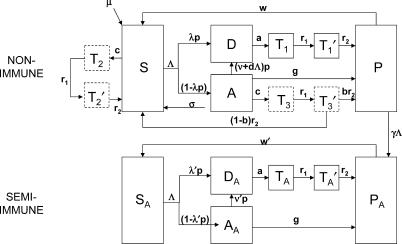
Schematic Diagram of Model of Malaria Illness and Treatment Drug use patterns in symptomatic, asymptomatic, and disease-free individuals.

Drug-sensitive parasites (RS) can infect susceptible hosts (S, S _a_), and a proportion *(q)* of untreated, infected hosts (A, A _a_). Partially resistant parasites (R1) can infect these hosts, and also those who have been treated and whose drug titer has declined, but not hosts whose immune responses have been stimulated by an infection immediately prior to or concurrent with treatment ( Figures 1 and 2). For example, they cannot infect T _1_ or T _1_′, because these treated hosts are developing a temporary immune-protected state (P, P _a_): their heightened immune state will temporarily protect them as the drug protection wanes. Fully resistant parasites (R2) can infect untreated hosts and any who have been treated, regardless of the drug titer, but not hosts whose immune systems are stimulated by a concurrent infection (as described for R1 above).Thus, the model assumes a degree of superinfection—a single host can harbor mixtures of parasites (RS, R1, R2), restricted only by the concentration of drug in the blood. However, in accord with common practice [ 11, 22, 23], we neglected intrahost dynamics and assumed independence of genetically distinct coinfecting parasites.

The lifetime of an infection is either the inverse of the rate at which the parasites are cleared by the immune system (1/ *g*) or the rate at which they are cleared by the drug (1/ *a*) following treatment. The average lifetime of an infection in a host population (ℓ̄) is the average of the lifetime of an infection in nonimmunes and the average lifetime in semi-immunes weighted by the proportion of infections that occur in each ( Equation 1C). The lifetime of an infection in nonimmunes (ℓ̄ *_NI_*) is equal to the fraction of infections that are treated multiplied by the rate of clearance by drug treatment plus the fraction of infections that are not treated multiplied by the rate of clearance by immune mechanisms ( Equation 1A). A similar formula ( Equation 1B) describes the lifetime of an infection in semi-immune individuals (ℓ̄ *_SI_*).



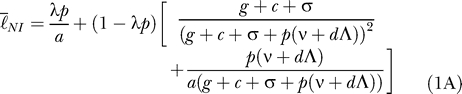





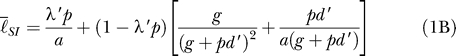





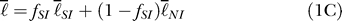



The lifetime of a fully resistant infection is always equal to the length of time required for the immune system to clear the infection (1/ *g*).

The relative fitness *(F)* is defined here as the ratio of the more resistant parasite type over the more sensitive type:



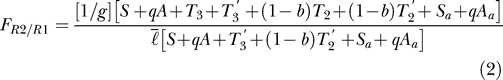





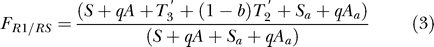



(It is interesting to note that Equation 3 is identical in structure to Equation 5 in [ 24], where *d*_r_ represents the hosts with residual drug [= T _3_′ + (1 − *b*) T _2_′], and *d*_s_ represents the host with no residual drug [= S + qA + S _a_ + qA _a_]. Although these equations were arrived at by different methods, and the variables S, A, T _3_, etc… in our model reflect changes in the underlying epidemiology, the qualitative results from the two approaches are similar.)

The fitness ratio will always be greater than or equal to one. We assumed that there is no significant cost of resistance. If the fitness ratio is greater than one, then the more resistant parasites have a competitive advantage over, or a greater fitness than, the more sensitive parasites. Assuming that sensitive parasites are initially established in the host population and a small fraction of resistant parasites arise, the fitness ratio minus one gives the fraction of spread of the more resistant parasites per generation. The quantity multiplied by 100 gives the percent of spread per parasite generation.



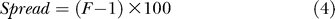



It has been observed, and is assumed here, that development of complete resistance to a drug requires multiple changes and is therefore stepwise. The appearance of R2 resistant parasites will always be preceded by R1 resistance.

### Combining Host and Parasite Model

To compute the fitness of partially and fully resistant parasites under different scenarios, we solve the equations describing disease patterns and human dosing for their equilibrium values and use the values of the state variables (S, A, D, etc…) in the fitness equations (Equations 2 and 3). High transmission is defined as one infectious bite per person per d (annual EIR = 365) and low transmission as 0.01 bites per person per d (annual EIR = 4). Life expectancy in this model is assumed to be 50 y; an individual remains nonimmune for the first 1.3 y of life in high transmission and 40 y in low transmission, after which they become semi-immune. Other parameter values are listed in Table 2. It is not well understood how immunity to malaria is acquired or how long it lasts. For the purposes of our model, however, sensitivity analysis suggests that the duration of 1/ *w* and 1/ *w*′ have a negligible effect on fitness, and so do not appreciably affect our results on the relative spread of resistance.

**Table 2:**
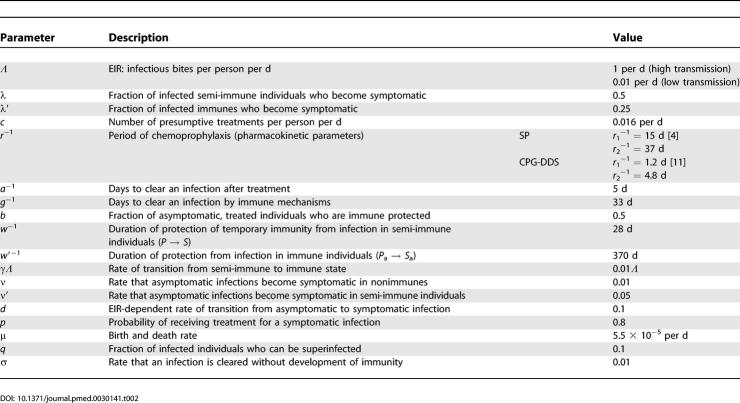
Parameters, Definitions, and Values

## Results

### Spread of Resistance in Low- versus High-Transmission Settings

**Figure 3:**
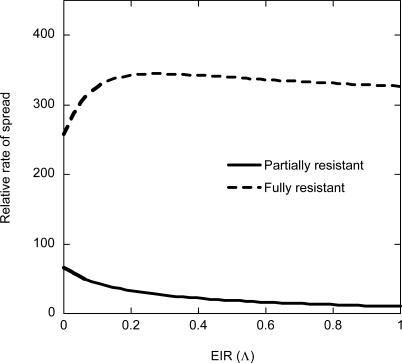
Effect of Increasing the Entomological Inoculation Rate *(Λ)* on the Percent Increase of R1 over RS or R2 over R1 Resistant Parasites

The fitness of partially resistant parasites (R1) decreases as transmission intensity *(Λ)* increases ( Figure 3). However, the fitness of R2 (fully resistant) parasites per generation initially increases for low to moderate values of *Λ* and then remains constant for moderate to high values of *Λ*. As *Λ* increases, the fraction of semi-immune individuals in the population increases because the transition from nonimmune to semi-immune is proportional to *Λ*. As the fraction of the population that is semi-immune increases, the potential of partially resistant parasites to spread decreases. However, as *Λ* increases, the number of infections increases, and drug use increases, leading to accelerated spread of fully resistant parasites.

### Effect of IPT on Spread of Resistance

**Figure 4:**
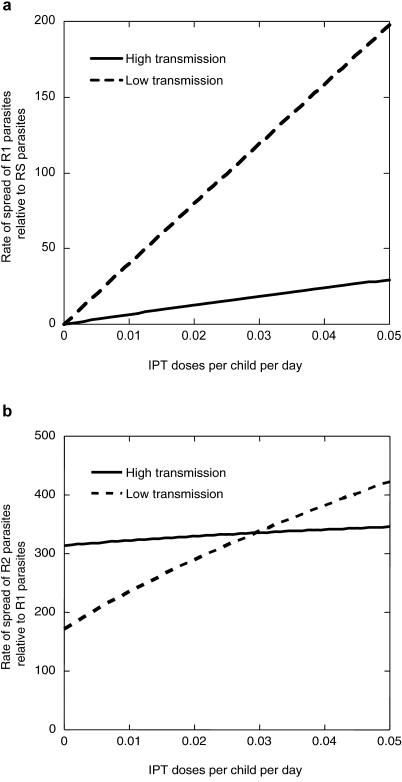
Effect of Increasing IPT Coverage with SP on the Percent Increase of R1 Relative to RS or R2 Relative to R1 Resistance Treatment effect is shown for percent increase of (A) R1 relative to RS or (B) R2 relative to R1 resistance. Increasing treatments of non-diseased individuals increases the relative spread of R1 and R2 resistance in low-transmission conditions (A and B) but only R1 resistance in high-transmission conditions (A).

To determine how IPT affects the spread of drug resistance, we evaluated the spread of partially and fully resistant parasites as a function of *c,* the number of doses of drug administered to nonimmune (S, A, P) individuals per unit time. As expected, the potential spread of resistance generally increases with dosing ( Figure 4). Surprisingly, in high-transmission areas the spread of full (R2) relative to partial (R1) resistance was unaffected by changes in IPT coverage ( Figure 4B). Furthermore, although the potential spread of R1 parasites increased with the rate of IPT dosing, the increase was much less pronounced in the high-transmission than the low-transmission setting ( Figure 4A). In quantitative terms, relative to RS, R1 parasites have the potential to increase by 65% each generation when EIR is low ( *Λ* = 0.01), but only 10% when EIR is high ( *Λ* = 1), assuming a presumptive dosing frequency of about one dose every 60 d ( *c* = 0.016 per d). This difference is influenced by the larger proportion of the population residing in the nonimmune categories in low compared to high transmission ( Table 1). The potential spread of partial resistance *per dose* of drug given to *nonimmunes* is identical in each scenario. In other words, when presumptive treatment is applied at a uniform rate across the population, then partial resistance spreads much more quickly in low-transmission conditions. However, when presumptive treatment is targeted to a specific subset of the nonimmune category such that the number of doses administered is equivalent, then the potential for partial resistance to spread is equivalent.

Of much greater concern, R2 parasites have about a 250-fold advantage over R1 parasites in low-transmission conditions; in high-transmission conditions, R2 parasites have a 300-fold advantage, whatever the value of *c*. The model also predicts that under conditions of perfect drug use, that is, with perfect diagnosis and compliance and no treatment of uninfected individuals, partial resistance would never spread.

### Effect of Drug Elimination Time on Spread of Resistance

**Figure 5:**
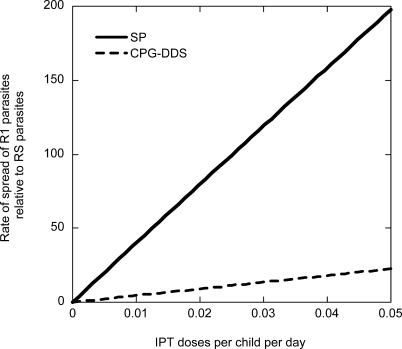
Effect of IPT Treatments with Drugs with Different Half-Lives (CPG-DDS or SP) on the Percent Increase of R1 Relative to RS in a Low-Transmission Setting Drugs with shorter half-lives (i.e., CPG-DDS) reduce the window of selective advantage of more resistant parasites.

Pyrimethamine and sulfadoxine in SP have elimination half-lives of 116 and 81 h [ 25], respectively, whereas dapsone and chlorcycloguanil (the active metabolite of chlorproguanil) in chlorproguanil-dapsone (CPG-DDS or Lapdap) have much shorter half-lives—about 12 and 20 h, respectively [ 26]. Because our model incorporates pharmacokinetic properties, it allows us to compare drugs with different clearance times. Compared to SP, the use of CPG-DDS for IPTi and treatment decreases the potential spread of partial resistance, in both low- ( Figure 5) and high-transmission conditions. In low-transmission conditions, CPG-DDS use also reduced the spread of R2 resistance, but to a lesser extent than for R1 parasites. The difference between SP and CPG-DDS in the spread of R2 in high-transmission conditions was negligible.

### Effect of Treatment

**Figure 6:**
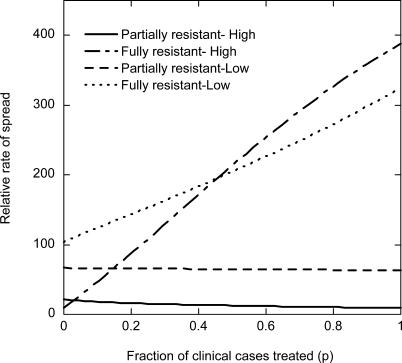
Effect on the Spread of R1 and R2 Resistance of Increasing the Proportion of Infected Individuals Who Are Treated Increasing drug usage in cases of true infection does not affect the spread of R1 but can select for R2 parasites if they are already present.

Overall, the spread of partial (R1) resistance is driven by drug treatment of uninfected, nonimmune individuals ( Figure 4). The dominant factors in the spread of full R2 resistance are the long duration of infections, which are refractory to drug treatment, and the greater proportion of human hosts available to R2 phenotypes. As the fraction of infections that are treated increases, the average lifetime of drug-susceptible and partially resistant infections decreases, but the lifetime of fully resistant infections remains the same. As a result, the fitness of the fully resistant parasites (R2) relative to the partially resistant parasites increases as the probability of being treated, *p,* increases ( Figure 6), but the spread of R1 is largely independent of *p*. Previous analyses [ 11, 24], based solely on drug use and drug pharmacokinetic properties, also identified the level of overall drug use (in infected and uninfected individuals) as the crucial factor for spread of partially resistant parasites, and the proportion of true infections treated with drug as the driving force for spread of fully resistant parasites.

### IPT in Adults versus Infants

**Figure 7:**
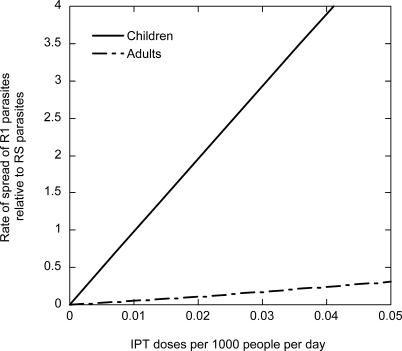
Effect of IPT in Adults or Infants on the Percent Increase of R1 Relative to RS Parasites Doses of drug administered to nonimmune infants have a greater impact on the spread of partial resistance.

We adjusted the model to reflect a drug use scenario in which semi-immune individuals rather than nonimmunes are treated presumptively, that is, in which individuals in categories S _a_, P _a_, and A _a_, rather than S, P, and A, are treated at regular intervals (rate *c*) with an antimalarial drug under high-transmission conditions. The potential spread of R1 parasites relative to RS parasites per dose of SP is much greater when IPT is implemented in infants than when it is implemented in adults, for example IPT during pregnancy ( Figure 7).

## Discussion

By combining a model of host disease and treatment with a model of relative parasite fitness, we are able to describe the effects of individual drug treatment on the population dynamics of malaria parasites. In this composite model, we incorporated two types of selection of resistant parasites—within-host selection and transmission selection. Within-host selection occurs when newly inoculated or surviving resistant parasites have an advantage over, and out-compete, drug-sensitive parasites within an individual who has received drug therapy. This selection is accounted for by incorporating the pharmacokinetics of drug treatment. Transmission selection occurs when resistant parasites, present due to in-host selection, are selectively transmitted and have a greater number of hosts available for the next generation. This type of selection is described by the differences in hosts available for fully resistant, partially resistant, and drug-sensitive parasites.

Despite the very high usage of chloroquine in high-transmission areas of Africa, resistance to chloroquine appeared much later than in lower-transmission areas such as Southeast Asia [ 27]. Once resistance appeared, it spread very quickly. Our model captures this behavior. Partially resistant parasites spread more slowly when infectious bites are more frequent. Fully resistant parasites have the potential to spread much more quickly than partially resistant parasites, regardless of the transmission intensity. Assuming that resistance is acquired in a stepwise manner, this is consistent with the observation that, in regions with high transmission rates, initial resistance spread more slowly and was detected later, followed by a rapid spread of highly resistant parasites. The high levels of acquired antimalarial immunity in sub-Saharan Africa, relative to lower transmission areas of South America and Southeast Asia, may be partly responsible for the delayed appearance of resistance in Africa.

Several recent studies have attempted to define the relationship between transmission intensity and spread of resistance [ 28– 30]. While the relationship is not completely clear, data collected from several villages in Uganda [ 28, 29] show that low and high levels of parasite resistance (R1 and RIII according to the WHO classification of parasitological response [ 31]) have opposite trends with respect to transmission intensity, consistent with our results. In the same study, no measure of resistance to SP was seen to decrease with increasing transmission intensity, contrary to our prediction that spread of partial resistance would decrease with increasing EIR. However, it is not at all clear how to relate the endpoints measured in the study to the partial and complete resistance discussed here, or how long ago resistance had been introduced into each village. The relationship is further confounded by significantly higher antimalarial drug consumption at lower transmission intensities.

In our model, transmission intensity (EIR) influences the incidence of disease, and therefore the number of doses of drug, as well as the development of immunity. These two factors have opposite effects on the spread of resistance, and we have captured this in our model. Hastings et al. [ 11] predicted that low-transmission conditions would favor the spread of complete resistance based on the assumption that the lifetime of an infection would be shorter in semi-immune individuals and that more infections were likely to be symptomatic and treated in conditions of low transmission. We assumed that the frequency of infection and clinical attack is lower in semi-immune individuals, but the lifetime of an untreated infection, *g,* is constant, based on the observation that semi-immune individuals can harbor long-lived asymptomatic infections.

Development of partial resistance, or drug tolerance, is a crucial step in the evolution of drug resistance [ 32] that has largely been ignored, particularly in mathematical models of drug resistance. Our model explicitly considers the intermediate development of drug tolerance on the spread of drug-resistant malaria. In both low- and high-transmission settings, as the number of disease-free, nonimmune people who are treated with drug (IPT) increases, the spread of partial resistance increases ( Figure 4), but the spread of fully resistant parasites in conditions of high transmission remains stable. Therefore, if some partial resistance to a particular drug has already been observed in a high-transmission setting, IPT is not likely to affect the spread of full resistance, although the subsequent spread is likely to be rapid. If no resistance to a drug has been measured, using that drug for IPT could accelerate the appearance of partial resistance, which could be followed by explosive spread of full resistance. Under conditions of low transmission, IPT could increase the spread of both partially and fully resistant parasites.

The pharmacokinetic properties of a drug should be taken into consideration when choosing a drug for IPTi. While drugs with long half-lives are desirable to maximize the period of protection from each treatment, the same property will also maximize the window for selection for resistant phenotypes [ 4]. The model shows that resistance can spread more quickly when a drug with a long half-life, such as SP, is used than when a drug with a shorter half-life such as CPG-DDS is used, although we do not account for the possibility of reduced efficacy of IPTi when using a drug with a shorter protective window. This result is both intuitive and consistent with previous observations and predictions. For example, Hastings et al. [ 11] predicted a relationship between human drug treatment rate and spread of partially resistant parasites similar to the results of our model for IPTi. Furthermore, they predicted that the impact of drug half-life on the transition from partial to complete resistance is very small compared to its impact on the transition from sensitive to partial resistance, similar to what we describe here for high-transmission conditions. It should be noted, however, that neither model accounts for the different effects of drugs on the sexual, transmissible blood forms of the parasite, but rather assumes that sexual stages are directly proportional to the asexual forms.

We hypothesized that administering drugs to disease-free individuals with little or no malaria-specific immunity, such as IPT during infancy, would have a greater impact on the spread of drug resistance than administering drugs to disease-free adults with some previous exposure and immunity, such as mass drug administration and IPT in pregnancy. The model predicts that partially resistant parasites have the potential to spread considerably faster when the same number of IPT doses is administered to infants (nonimmunes) compared to adults (semi-immune individuals). We did not explicitly consider presumptive drug use outside of IPT, and it is not clear whether drug use within IPTi would be overshadowed by general presumptive treatment. However, many of these conclusions can be extrapolated to presumptive treatment, including the prediction that presumptive treatment in nonimmunes will contribute more significantly to the spread of drug resistance than that in semi-immune individuals.

These results show the potential effect of IPT on the spread of drug-resistant parasites in low- and high-transmission areas. These predictions are not a quantitative evaluation of the inevitable impact of IPTi, but rather a qualitative assessment of the relative potential of partially and fully resistant parasites to establish in a community of available hosts under different drug use strategies. The mathematical analysis permits our understanding and assumptions about the problem to be made explicit and be evaluated. The model can be used as a tool to determine which are the most critical questions to be answered before IPTi is implemented on a broad scale. Although our model captures the factors influencing the epidemiology of drug-resistant malaria more thoroughly than its predecessors, its results, like those of any model, are sensitive to its underlying structure and should be used to define research priorities rather than to accept or abandon specific programs.

This analysis highlights the importance of carefully selecting the drug to be used in IPTi programs. Cross-sectional surveys to determine the existing levels of resistance to a particular drug should be undertaken prior to introducing IPT programs for infants. Drugs to which little or no resistance exists are not advisable for IPT in high-transmission areas, but IPTi is not likely to significantly impact the spread of highly resistant parasites in areas where partial resistance is already established. Drugs with shorter half-lives should be considered, and the duration of protection from infection should be weighed against the opportunity for spread of resistance.

## Supporting Information

Text S1 Human Dosing Model Equations Differential equations for the human dosing model describing the relationships between infection, disease, treatment, and immunity.
(37 KB DOC)

**Patient Summary**

**Background**

Malaria has been on the rise in the past decade, mainly because the parasites that cause the disease have become resistant to the widely used antimalarial drugs. There is no resistance yet to a promising new class of drugs, called artemisinins. While neither malaria eradication nor a vaccine seem feasible goals in the next few years, efforts to combat malaria have recently gained momentum. One strategy under consideration is to treat people before they get sick. Such treatment does not prevent infection—which happens through mosquito bites—but it can prevent disease by eliminating the parasites once they get into the blood. Researchers have some experience with prevention strategies in pregnant women and infants, who are particularly vulnerable to malaria. In most studies, the participants received intermittent preventive treatment (IPT), which means women received two or three courses of treatment in the second and third trimesters, and infants got three courses at 2, 3, and 9 months, together with routine childhood immunizations.

**Why Was This Study Done?**

IPT has been found to reduce the devastating consequences of malaria during pregnancy and infancy, provided that the drugs used are still effective (i.e., that the local parasites have not developed resistance). As a consequence, many people are calling for IPT to be used more widely, and there is even discussion of expanding preventive treatment beyond pregnant women and infants. But experts have warned that widespread treatment of healthy individuals will increase drug resistance in the parasite, and that the potential risks and benefits of IPT need to be weighed carefully. One way to do this is to use predictive disease modeling to estimate the health benefits as well as the risks—especially drug resistance—under various scenarios. The authors of this study have focused on estimating the impact of IPT in adults and infants on parasite drug resistance. It is important to distinguish infants and adults, because resistance is more likely to develop in infants whose immune system is immature. In addition, the blood of infants contains higher levels of parasites, which means resistant parasites are more likely to be picked up by mosquitoes and spread to others.

**What Did the Researchers Do and Find?**

They used a mathematical model that could take into account several different variables. A number of them influenced the development of resistance, including transmission frequency (i.e., the number of mosquito bites that transmit malaria parasites per individual per year), the existing level of resistance to the drug of choice, characteristics of the drug (specifically, how quickly or slowly it is broken down or removed from the body), and treatment frequency (i.e., the number of drug courses per year). The model predicts that partially resistant parasites are more likely to emerge and spread in low-transmission areas and fully resistant ones in high-transmission areas. IPT in infants could accelerate the spread of resistant parasites. This is more likely when drugs that are slowly broken down or removed are used, because that prolongs the time window during which parasites are exposed to low levels of drugs that favor selection of resistant variants.

**What Does This Mean?**

The researchers warn that the use of IPT drugs to which little or no resistance exists (such as the artemisinins) will likely accelerate resistance development and should be avoided, especially in high-transmission areas. For IPT in infants, they suggest that drugs should be chosen that are broken down or removed quickly and to which local resistance levels are low. However, like all other modelling efforts, this one can only suggest likely outcomes and is best used as a tool to define research priorities and highlight the most critical questions that must be answered before the widespread implementation of a particular strategy.

**Where Can I Find More Information Online?**

Pages from the US Centers for Disease Control and Prevention:

http://www.cdc.gov/malaria/control_prevention/control.htm

WHO pages on malaria, which contain a section on malaria and pregnancy:

http://www.who.int/topics/malaria/en

MedlinePlus pages on malaria:

http://www.nlm.nih.gov/medlineplus/malaria.html

UNICEF page on malaria with links:

http://www.childinfo.org/eddb/Malaria

IPTi Consortium:

http://www.ipti-malaria.org

## Abbreviations

CPG-DDS: chlorproguanil-dapsone
EIR: entomological inoculation rate
IPT: intermittent preventive treatment
IPTi: IPT for infants
SP: sulfadoxine-pyrimethamine
