# Supplementary material for: Potential Impact of Intermittent Preventive Treatment (IPT) on Spread of Drug-Resistant Malaria
Source: PLoS Med. 2006 Apr 4;3(5):e141. doi: 10.1371/journal.pmed.0030141 (PMC1440294; doi:10.1371/journal.pmed.0030141)
Supplement: Supplementary file 1 [file pmed.0030141.sd001.doc]

Supporting Text 1: Human dosing model equations

$\begin{aligned} \frac{\text{dS}}{\text{dt}}|_{t=\infty}=\mu-\Lambda S+\omega P+\sigma A-\text{cS}+(1-b)r_{2}T_{3^{'}}-\mu S \\ \frac{\text{dD}}{\text{dt}}|_{t=\infty}=\lambda p\Lambda S-\text{aD}+(\nu+d\Lambda)\text{pA}-\mu D \\ \frac{\text{dA}}{\text{dt}}|_{t=\infty}=(1-\lambda p)\Lambda S-\text{gA}-\sigma A-\text{cA}-(\nu+d\Lambda)\text{pA}-\mu A \\ \frac{\text{dT}_{1}}{\text{dt}}|_{t=\infty}=\text{aD}-r_{1}T_{1}-{\mu T}_{1} \\ \frac{\text{dT}_{1^{'}}}{\text{dt}}|_{t=\infty}=r_{1}T_{1}-r_{2}T_{1^{'}}-{\mu T}_{1^{'}} \\ \frac{\text{dT}_{2}}{\text{dt}}|_{t=\infty}=\text{cS}-r_{1}T_{2}-{\mu T}_{2} \\ \frac{\text{dT}_{2^{'}}}{\text{dt}}|_{t=\infty}=r_{1}T_{2}-r_{2}T_{2^{'}}-{\mu T}_{2^{'}} \\ \frac{\text{dT}_{3}}{\text{dt}}|_{t=\infty}=\text{cA}-r_{1}T_{3}-{\mu T}_{3} \\ \frac{\text{dT}_{3^{'}}}{\text{dt}}|_{t=\infty}=r_{1}T_{3}-r_{2}T_{3^{'}}-{\mu T}_{3^{'}} \\ \frac{\text{dP}}{\text{dt}}|_{t=\infty}=\text{gA}+r_{2}T_{1^{'}}+\text{br}_{2}T_{3^{'}}-\omega P-\gamma\Lambda P-\mu P \\ \frac{\text{dS}_{a}}{\text{dt}}|_{t=\infty}={\omega P}_{a}-{\Lambda S}_{a}-{\mu S}_{a} \\ \frac{\text{dD}_{a}}{\text{dt}}|_{t=\infty}=\lambda^{'}{p\Lambda S}_{a}-\text{aD}_{a}+\nu^{'}A_{a}-{\mu D}_{a} \\ \frac{\text{dA}_{a}}{\text{dt}}|_{t=\infty}=(1-\lambda^{'}p){\Lambda S}_{a}-\text{gA}_{a}-\nu^{'}A_{a}-{\mu A}_{a} \\ \frac{\text{dT}_{a}}{\text{dt}}|_{t=\infty}=\text{aD}_{a}-r_{1}T_{a}-{\mu T}_{a} \\ \frac{\text{dT}_{a^{'}}}{\text{dt}}|_{t=\infty}=r_{1}T_{a}-r_{2}T_{a^{'}}-{\mu T}_{a^{'}} \\ \frac{\text{dP}_{a}}{\text{dt}}|_{t=\infty}=r_{2}T_{a^{'}}+\text{gA}_{a}-{\omega P}_{a}-{\mu P}_{a} \end{aligned}$
